# Impact of age on the transmission of SARS-CoV-2 in healthcare workers: Influence of nonoccupational risk factors

**DOI:** 10.1007/s00508-024-02346-0

**Published:** 2024-04-08

**Authors:** Luis Corral-Gudino, María Piedad Del-Amo-Merino, Jésica Abadía-Otero, Irene Merino-Velasco, Yolanda Lorenzo-Fernández, Jesús García-Cruces-Méndez, José María Eiros-Bouza, Marta Domínguez-Gil González

**Affiliations:** 1 Department of Internal Medicine, Dermatology and Toxicology. Hospital Universitario Rio Hortega, Valladolid. School of Medicine, Universidad de Valladolid, Avda. Ramón y Cajal, 7, 47005 Valladolid, Spain; 2 Occupational Risk Prevention Service, Hospital Universitario Río Hortega, Gerencia Regional de Salud de Castilla y Leon (SACYL), C/Dulzaina n°2, 47012 Valladolid, Spain; 3 Department of Internal Medicine, Hospital Universitario Río Hortega, Gerencia Regional de Salud de Castilla y Leon (SACYL), C/Dulzaina n°2, 47012 Valladolid, Spain; 4 Department of Microbiology, Hospital Universitario Río Hortega Universidad de Valladolid, Gerencia Regional de Salud de Castilla y Leon (SACYL), C/Dulzaina n°2, 47012 Valladolid, Spain; 5 Occupational Risk Prevention Service, Hospital Universitario Río Hortega, Gerencia Regional de Salud de Castilla y Leon (SACYL), C/Dulzaina n°2, 47012 Valladolid, Spain; 6 Department of Preventive Medicine and Hospital Epidemiology, Hospital Universitario Río Hortega, Gerencia Regional de Salud de Castilla y Leon (SACYL), C/Dulzaina n°2, 47012 Valladolid, Spain; 7 Department of Microbiology, Hospital Universitario Rio Hortega, Valladolid. School of Medicine, Universidad de Valladolid, Avda. Ramón y Cajal, 7, 47005 Valladolid, Spain

**Keywords:** COVID-19, Vaccination, Young people, Social distance, Health personnel

## Abstract

**Background:**

The incidence of Severe Acute Respiratory Syndrome Coronavirus 2 (SARS-CoV‑2) infection was highest among older adults early in the COVID-19 pandemic; however, this pattern was later reversed with young adults showing the highest incidence. The aim of this study was to identify risk factors in healthcare workers (HCWs) associated with this evolution.

**Methods:**

We conducted a survey nested within a prospective cohort study of 680 HCWs from a tertiary referral public hospital who received 2 doses of SARS-CoV‑2 vaccine in January and February 2021 (VACCICO-VAO cohort). In October 2022 all participants were invited to participate in a survey. Risk factors were tested for association with COVID-19 ever, the number of COVID-19 episodes, and the time to the first episode.

**Results:**

Among 350 respondents (51% response rate, 90% female, mean age 48.1 years), 323 COVID-19 episodes were diagnosed during the study period. Multivariable analysis revealed that age < 35 years vs. > 50 years (odds ratio, OR 2.12, 95% confidence interval, CI 1.27–3.51; *P* = 0.004) and not maintaining social distance at social events (OR: 1.82, 95% CI: 1.16–3.19; *P* = 0.011) were associated with a higher risk of COVID-19. Age < 35 years (hazard ratio, HR 1.70, 95% CI 1.14–2.54; *P* = 0.010), and not maintaining social distance (HR 1.34, 95% CI 1.05–1.72; *P* = 0.020) were also associated with the time to the first episode.

**Conclusions:**

The youngest HCWs had the highest incidence rate of COVID-19, which was not explained by occupational risk factors or health conditions. The increase in nonoccupational exposure since the end of the lockdowns in summer 2020 could by a key factor.

**Graphic abstract:**

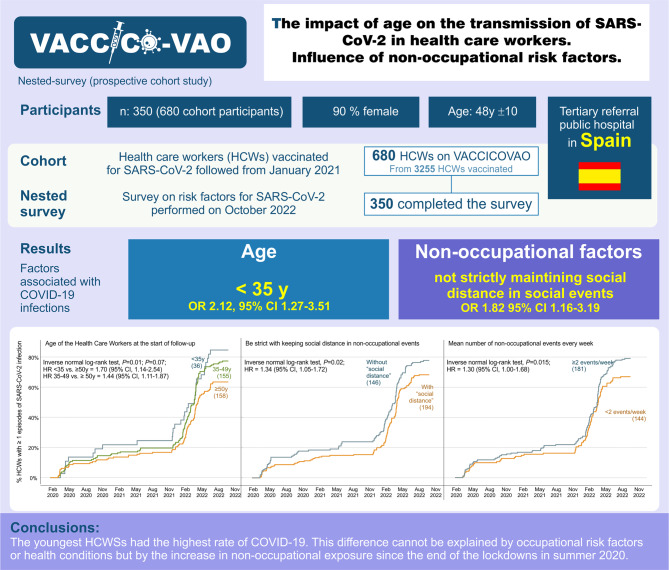

## Introduction

Early in the coronavirus disease 2019 (COVID-19) pandemic (January–May 2020) it was observed that older adults were more vulnerable to infections with a higher incidence [1], more severe outcomes, and higher case fatality rates [2] than younger individuals; however, after July 2020 this pattern was reversed and the incidence was higher in the younger adults than in older adults [3]. Recent studies have confirmed an increased rate of COVID-19 in young people in the general population [4].

An apparent age paradox in the rate of severe acute respiratory syndrome coronavirus 2 (SARS-CoV-2) infection in healthcare workers (HCWs) was also demonstrated [5]. The HCWs are at a higher risk of SARS-CoV‑2 infection, because their role is to take care of infected patients [6, 7]. Frontline HCWs are at the highest risk of acquiring COVID-19 [8, 9]. The highest serological prevalence rates of all the population groups during the first months of the COVID-19 pandemic in Spain [10] was among HCWs (10%). Spain reported the highest cumulative number of cases of COVID-19 in HCWs worldwide during the first year of the pandemic [11]. The prevalence of SARS-CoV 2 infections in HCWs varies greatly between countries, with the highest prevalence of SARS-CoV‑2 infection in HCWs in Spain in May 2020 (20%) [10], Italy (18%) [12], and the USA (27%) [13], and a low prevalence of 4.2% reported in China [14].

Several risk factors were associated with the occupational transmission of SARS-CoV‑2 in HCWs at the start of the pandemic, including the lack or inappropriate use of personal protective equipment (PPE), working directly with infected patients, exposure to aerosol-generating procedures (AGPs), excessive workload, long working hours, sleep deprivation, and lack of training and education in infection prevention and control [6, 15, 16]. With the evolution of the pandemic, risk factors such as working directly with patients with COVID-19 or performing AGPs were no longer associated with an increase in SARS-CoV‑2 infections, supporting the efficacy of compliance with PPE and increased staff awareness and experience [17, 18]. Even those who worked with infected patients in intensive care units had a lower risk of infection than those who did not [19]. In addition, with the start of the massive HCW vaccination against SARS-CoV‑2 in 2021, there has been a dramatic decrease in disease severity; however, this decrease in disease severity was not accompanied by a decrease in the number of cases, and there was a striking increase in the incidence of SARS-CoV‑2 infections with new variants [20]. With a clear decrease in occupational transmission, the reason for the continued number of incident SARS-CoV‑2 infection cases in HCWs may be explained by nonoccupational risk factors, which play a major role in community and household exposure [17].

The study aimed to assess the role of nonoccupational risk factors in the risk for SARS-CoV‑2 infections or reinfections in a HCWs cohort and to test the hypothesis that these factors may be more important than occupational factors in the current transmission of COVID-19.

## Patients, material and methods

A cross-sectional study nested within the VACCICO-VAO prospective cohort study was performed. The characteristics of the VACCICO-VAO cohort have been described previously [21]. In summary, the Hospital Universitario Rio Hortega (HURH) in Valladolid, Spain, vaccinated all HCWs between January and March 2021. The HCWs were invited to participate in a prospective cohort study. As a part of the cohort follow-up, from October to December 2022 (coinciding with the administration of the fourth dose of SARS-CoV‑2 vaccine), all participants were invited to complete a survey on the risk factors for SARS-CoV‑2 infection during the pandemic period. The cohort study was approved by the Ethics Committee for Drug Research (CEIm) of the HURH (protocol number: CEIC 21-E0031).

### Participants and recruitment

All HCWs who were vaccinated against SARS-CoV-2 at the beginning of 2021 and were enrolled in the VACCICO-VAO cohort were identified. In October 2022, all participants in the VACCICO-VAO cohort were invited to participate in the survey. Two methods were used for survey recruitment: a) All participants in the VACCICO-VAO cohort were invited by email, and b) participants were reminded when they were vaccinated at the Occupational Risk Prevention Service.

### Measures

The survey contained two sections to measure occupational and nonoccupational risk factors. For the occupational risk factors, respondents were asked to identify their grade of exposure to SARS-CoV‑2 during the workday, which was classified into three categories: (i) very high: HCWs working in intensive care units, COVID wards or emergency wards; (ii) high: HCWs working in non-COVID hospital wards and operating rooms or performing complementary tests with air or digestive exposure (bronchoscopy, endoscopy, ear nose and throat and maxillofacial examinations) and (iii) intermediate: HCWs working in consultations, laboratories, administrative staff, pharmacies, dining rooms, and cafeterias or performing other complementary tests. The responders were also asked whether they worked directly and for long periods of time in rooms or spaces in which high-flow oxygen therapy or nebulization was administered, and whether they worked on night shifts or as 24-hour on-site guards.

For the nonoccupational risk factors, respondents were asked about their home characteristics and household members (number of people living in the home, number of people with direct exposure to SARS-CoV‑2, number of students), shared spaces at home, such as elevators or stairs, and location (rural, urban, peri-urban, and others). They were asked about their means of transportation (What is your most common means of transportation to the hospital?), and lifestyle (exposure to the virus outside the workplace). To collect data on social distancing, the participants were invited to answer two questions in this section: (1) how do you define yourself from the perspective of social contact outside the workplace? with three options: (i) little social contact, (ii) regular social contact but maintaining social distance or (iii) regular social contact with little or no social distance and (2) number of times per week that they attended social events, such as dinners, lunches, going out for snacks, theaters, concerts and gyms. For all questions, participants were asked to describe their actions over the previous year.

The demographic information included age, sex, ethnicity, job title, professional category, and comorbidities at the start of the study period. Data on vaccination status, SARS-CoV‑2 infections, and reinfections were prospectively collected during the cohort follow-up. Data on infection and reinfection were collected in the survey and double checked in the registry of the Occupational Risk Prevention Service. Data on vaccinations for other diseases (measles, tuberculosis, and influenza) were also included in the survey.

### Statistical analysis

Participants were grouped into three age groups (under 35 years, 35–49 years, and 50 years or older) to analyze the role of different risk factors according to age.

We compared the different risk factors for having a SARS-CoV‑2 infection at least once using simple logistic regression and the mean number of COVID-19 episodes using simple linear regression. Factors with statistically significant associations in the unadjusted model were included in the multivariable analysis. To assess the evolution of risk factors during the pandemic, we performed a Cox survival analysis. We use IBM SPSS Statistics for Windows, Version 26.0 (IBM Corp., Armonk, NY, USA) for the analysis.

## Results

A total of 350 members of the initial cohort of 680 participants (51%) completed the risk factor survey (90% females, age 48.1 years). The respondents comprised 185 nurses, 61 doctors, 34 administrative clerks, 15 physiotherapists, 11 hospital wardens and 44 with other job positions. During the study period 323 episodes of SARS-CoV‑2 infection were diagnosed among the 350 respondents: 61 from February 2020 to January 2021, 76 from February 2021 to January 2022, and 186 from February 2022 to November 2022. The main participant characteristics and vaccination status are shown in Table 1.

**Table 1:** Characteristics of VACCICO-VAO participants

Characteristics	HCWSs with no SARS-CoV‑2 infection (*n* = 94)	HCWSs with one case of reported SARS-CoV‑2 infection (*n* = 154)	HCWSs with more than one case of reported SARS-CoV‑2 infection (*n* = 102)
Gender (female, %)	86 (92%)	137 (89%)	93 (91%)
*Age (years)*			
< 35	5 (5%)	18 (12%)	12 (13%)
35–49	33 (35%)	70 (46%)	52 (51%)
≥ 50	56 (60%)	65 (42%)	37 (36%)
Mean, years, SD	51± 8	47 ± 10	46 ± 10
*Charlson comorbidity*			
Mean, SD	1.1 ± 1.4	0.8 ± 1.1	0.6 ± 0.9
*Professional category*			
Administrative	11 (12%)	13 (8%)	13 (13%)
Doctor	21 (22%)	31 (20%)	9 (9%)
Nurse	32 (34%)	64 (42%)	43 (42%)
NACT	13 (14%)	18 (12%)	15 (15%)
Physiotherapist	2 (2%)	5 (3%)	8 (8%)
Porter	1 (1%)	5 (3%)	5 (5%)
Other	14 (15%)	18 (12%)	9 (9%)
*Workplace*			
Intensive care	8 (9%)	11 (7%)	5 (5%)
Emergency room	4 (4%)	5 (3%)	5 (5%)
COVID-19 wards	8 (9%)	27 (18%)	18 (18%)
*Other wards or OP*			
DT risk COVID	29 (31%)	38 (25%)	26 (25%)
DT low risk	1 (1%)	1 (1%)	1 (1%)
Operating rooms	5 (5%)	21 (14%)	9 (9%)
Other	21 (22%)	25 (16%)	16 (16%)
	18 (19%)	26 (17%)	22 (22%)
*Vaccination*			
Vaccine (1st, 2nd)	94 (100%)	154 (100%)	102 (100%)
Moderna®	13 (14%)	26 (17%)	21 (21%)
Pfizer®	81 (86%)	128 (83%)	81 (79%)
Vaccine (3rd)	89 (95%)	149 (97%)	89 (87%)
Moderna®	14 (15%)	23 (15%)	18 (18%)
Pfizer®	75 (80%)	126 (82%)	71 (70%)
None	5 (5%)	5 (3%)	13 (13%)
3rd dose different brand	2 from 89 (2%)	8 from 149 (5%)	1 from 89 (1%)
*Vaccine (4th)*			
Pfizer®	54 (57%)	48 (31%)	34 (33%)
None	40 (43%)	106 (69%)	68 (67%)

*DT* diagnostic test, *HCWS* healthcare workers *NACT* Nursing Auxiliary Care Technician, *OP* outpatient clinics, *SARS-CoV‑2* Severe acute respiratory syndrome coronavirus 2, *COVID-19* Coronavirus disease 19

**Fig. 1:**
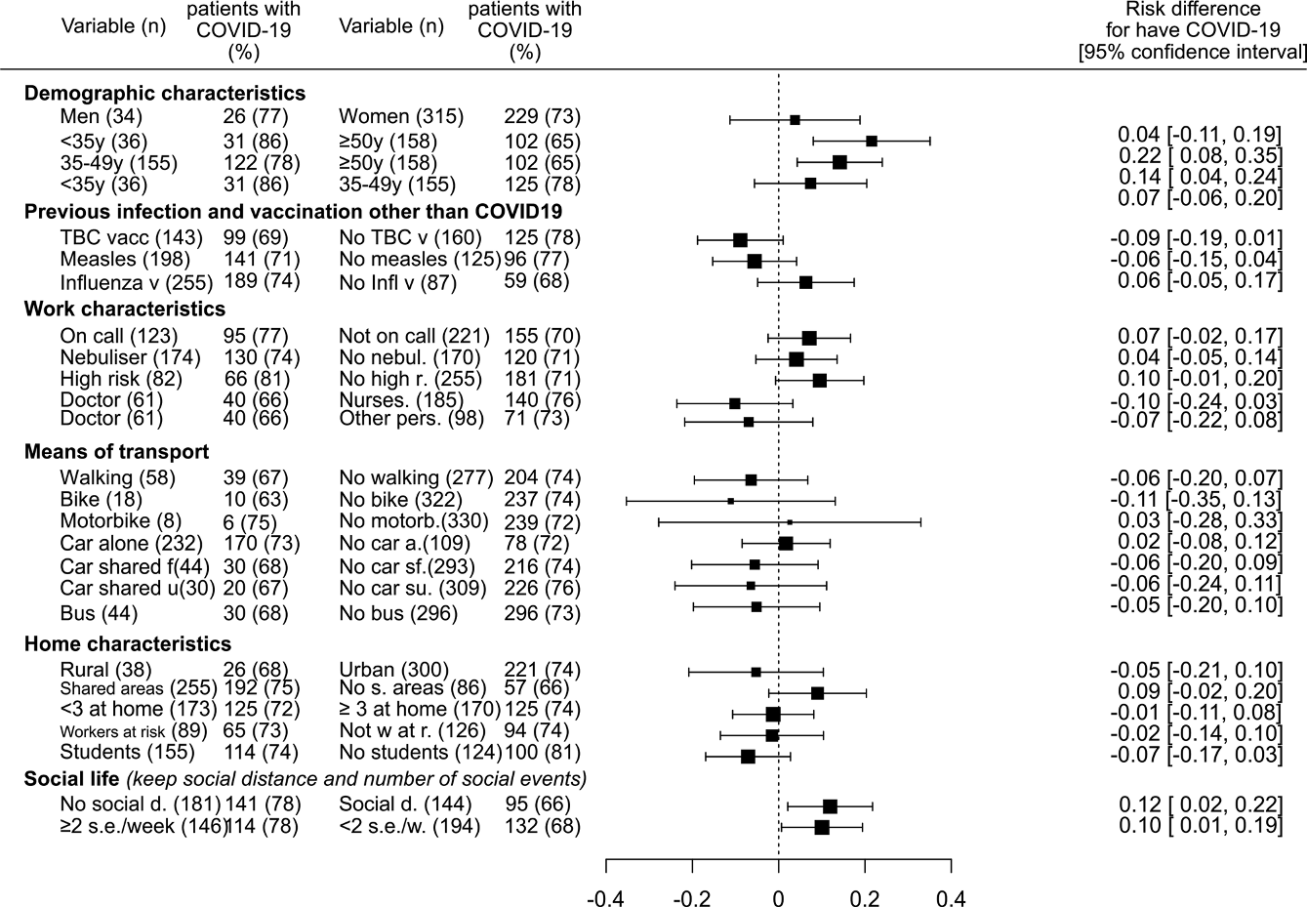
Forest plot of risk factors associated with at least one episode of Severe acute respiratory syndrome coronavirus 2 (SARS-CoV‑2) infection

Among the risk factors analyzed, age younger than 35 years vs. 50 years or older, not being strict about maintaining social distance at social events and having two or more nonoccupational social events per week were associated with a higher risk of having at least one episode of SARS-CoV‑2 infection (Fig. 1). In the multivariable analysis, two variables retained their association: age younger than 35 years (OR: 2.12, 95% CI: 1.27–3.51; *P* = 0.004) and not being strict about maintaining social distance at social events (OR: 1.82, 95% CI: 1.16–3.19; *P* = 0.011).

**Fig. 2:**
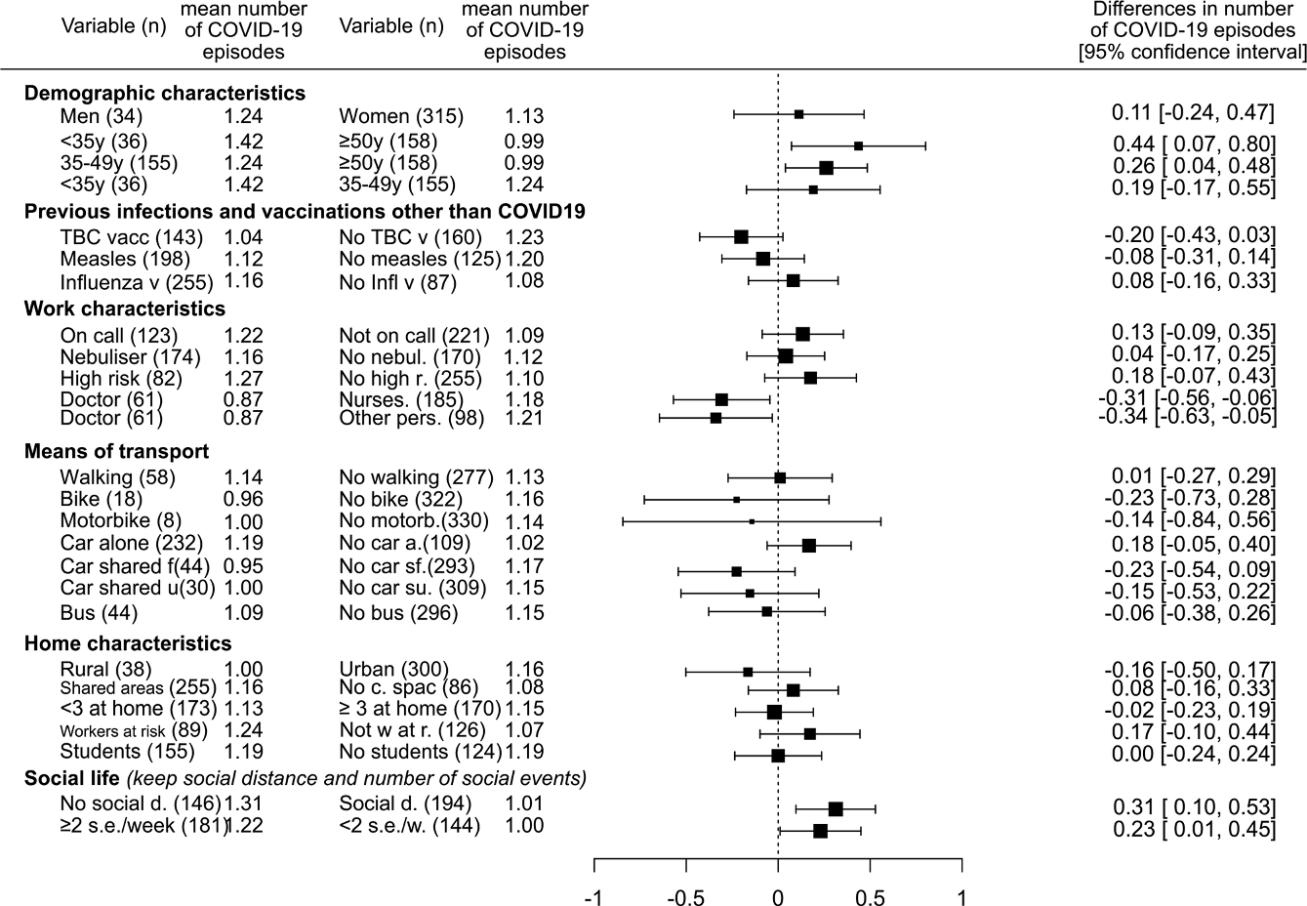
Forest plot of risk factors associated with number of Severe acute respiratory syndrome coronavirus 2 (SARS-CoV‑2) infections

From the risk factors analyzed for association with the number of COVID-19 infections, age younger than 50 years, being a nurse vs. a doctor, not being strict with maintaining social distance, and having fewer than two nonoccupational social events per week were associated with a low number of COVID-19 infections in the unadjusted analysis (Fig. 2). The variables associated with a higher mean number of COVID-19 episodes per person in the multivariable analysis were age < 35 years (*P* = 0.010), not being strict about maintaining social distance at social events (*P* = 0.023) and working as a nurse vs. as a doctor (*P* = 0.033).

**Fig. 3:**
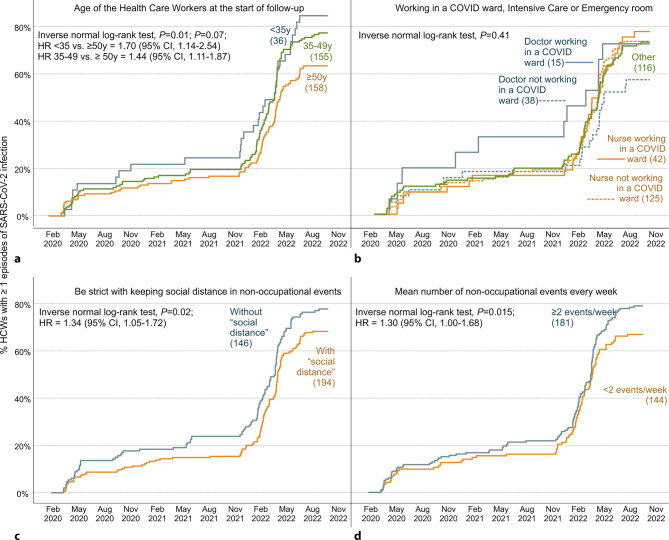
Survival analysis of the first COVID-19 infection in healthcare workers in the VACCICO-VAO cohort. **a** According to age, **b** workplace: COVID-19 wards, intensive care, or emergency room vs. other areas, **c** strict maintenance of social distance in social events, **d** mean number of nonoccupational events every week

Figure 3 shows the evolution of new cases of Coronavirus disease 19 (COVID-19) from February 2020 to November 2022 and the hazard ratio (HR) according to age (Fig. 3a), workplace (COVID-19 wards, intensive care, or emergency room vs. other areas) (Fig. 3b), and being strict about maintaining social distance at social events (Fig. 3c), and the mean number of nonoccupational events attended per week (Fig. 3d). Notably, during the pandemic evolution, the major differences between the curves corresponded with the summer period.

## Discussion

These results suggest that nonoccupational risk factors might have become the major risk factors for SARS-CoV‑2 infection in HCWs, replacing occupational risk factors. The effectiveness of the vaccination campaign, the natural immunity acquired after previous infection, the decreased severity of COVID-19 with the emergence of new SARS-CoV‑2 variants, and the lengthy duration of the pandemic have changed the perception of HCWs regarding how to protect themselves from SARS-CoV‑2 infection. In Spain, the level of concern regarding SARS-CoV‑2 infections in nonoccupational activities has decreased [22]. In our cohort, this could have led to a substantial increase in the number of HCWs infected with SARS-CoV‑2 in the first months of 2022.

We have previously shown an increase in the incidence of COVID-19 among younger HCWs [20], which could not be explained by occupational exposure or vaccination coverage. In this survey, we analyzed the implications of social life and community exposure to the virus in HCWs. Approximately two thirds of HCWs younger than 35 years (22 of 35, 63%) were not strict in maintaining social distance, whereas only half of HCWs aged 35–49 years (70 of 151, 46%) and one third of those aged 50 years and older (54 of 154, 35%) had this risk factor. In addition, the number of social activities per week was highest in the younger than 35 years age group and lowest in the 50 years and older age group. A systematic review by Chou et al. [18] showed no consistent association between age and risk of SARS-CoV‑2 infection. This difference can be explained by differences in the period during which the studies were conducted. The studies included in the systematic review included data from 2020 or even from the first months of the pandemic, when countries were in lockdown. In this study, the increase in cases in the youngest age group started in 2021, a period that is not represented in the studies included in the systematic review. Another likely explanation for the increase in cases after the summer of 2021 is that during the first months of 2021, the vaccine was better matched to the circulating strain.

**Fig. 4:**
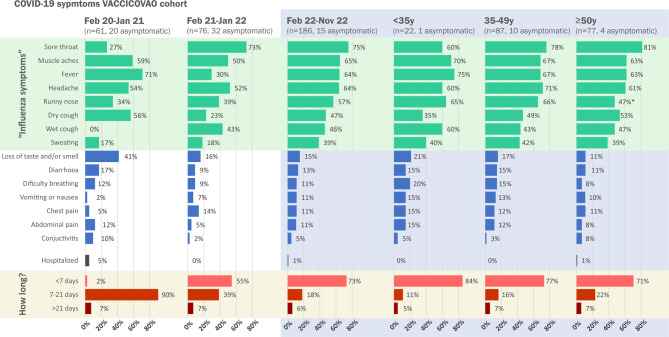
Changes in the clinical presentation of SARS-CoV‑2 infection in the VACCICOVAO cohort during the pandemic period

One of the differences between the periods that may have affected our outcomes is the low acceptance of vaccination with the 4th dose. During the pandemic, vaccination hesitancy among HCWs increased and has become a matter of concern [23, 24]. The effectiveness of COVID-19 vaccines and previous infections in reducing the severity of the disease, with a clear improvement in symptoms during the last periods, as evidenced by our study, with no clear difference by age (Fig. 4), is likely one of the main reasons for the increase in refusal to be vaccinated. This hesitancy against vaccination is more pronounced in young adults [25]; however, in our cohort there were no differences between age groups, which could explain the differences between age groups for COVID-19 infections (44% of participants in the under 35 years age group received the 4th dose of vaccine, compared with 35% in the 35–49 years age group and 46% of those age 50 years and older group, *P* = 0.171). In addition, the reduction in adherence to protective measures after vaccination [26] may have been a confounding factor in this cohort; however, this factor was not investigated in this study.

Regarding occupational factors, this study did not show any differences in risk of SARS-CoV‑2 infections between occupational groups. In seroprevalence studies, nurses have had a higher prevalence of SARS-CoV‑2 antibodies than doctors [27]. The small number of doctors included in this study could limited the power to detect statistically significant differences, although the number of COVID-19 episodes was lower in doctors. In addition, the lack of differences between workplaces [28] when comparing HCWs working in dedicated COVID-19 areas with other workers can be explained by the limited sample size.

## Limitations

This study has several limitations, some of which have been reported previously [20, 21]. One of the main drawbacks of the nested survey is the low response rate. This may have caused selection bias, especially considering that the participants who completed the survey may have been those most interested in the COVID-19 pandemic and may have been more willing to continue to receive additional doses of vaccine. The survey coincided with the administration of a fourth dose of SARS-CoV‑2 vaccine to HCWs. The delay or refusal to receive vaccination increased in HCWs with each additional dose [29]. All the participants in the cohort received the first two doses of vaccine in the first months of 2021, whereas more than half of the cohort refused to have a fourth dose. The HCWs in the cohort who decided to reject the fourth dose of vaccine may have chosen not to participate in the survey to avoid being questioned about their refusal; however, the survey results are likely to be representative of the cohort as the rate of vaccination acceptance was similar to that of HCWs in the hospital overall. In the hospital, of the 3255 HCWs who received the first two doses of vaccine from January to March 2021, 2710 (83%) agreed to receive the third dose in October 2021, and 1489 (46%) agreed to receive the fourth dose in October 2022.

Another limitation of our study is the influence of potential confounding factors that may have affected the risk estimates. The nested cross-sectional design of this study limited our ability to adjust for confounding factors. We attempted to minimize confounding by using multivariable models adjusting for potential confounding factors identified in the unadjusted analysis.

## Conclusion

This study shows that social exposure of HCWs likely played an important role in the transmission of SARS-CoV‑2 infection, possibly even greater than that of the occupational exposure. This finding supports the idea that reducing social exposure is a key factor in controlling pandemics. The rigorous national lockdowns established at the start of the pandemic have been questioned because of their tremendous impact on people’s physical and mental well-being and their social and economic impacts. Despite these clear disadvantages, many researchers believe that there is strong evidence that the first lockdowns played an essential role in decreasing the rate of new COVID-19 cases, saving lives, and preventing the collapse of national health systems. The major role that social risk factors played in the transmission of the disease towards the end of the study period confirms the importance of their control during the early stage of the pandemic. With a milder presentation of the disease, social risk factors currently have little impact on mortality rates or the burden of the health system; however, their control likely played an essential role in limiting the size of the first COVID-19 wave in 2020.

## Data Availability

The data that support the findings of this study are available from the corresponding author, Corral-Gudino, Luis, upon reasonable request.
